# Assessment of accumulation of heavy metals in soil, irrigation water, and vegetative parts of lettuce and cabbage grown along Wawan Rafi, Jigawa State, Nigeria

**DOI:** 10.1007/s10661-022-10360-w

**Published:** 2022-08-20

**Authors:** Balarabe Sarki Sagagi, Abdu Muhammad Bello, Haruna Abubakar Danyaya

**Affiliations:** 1 Department of Chemistry, Kano University of Science and Technology, Wudil, Kano State Nigeria; 2 Department of Science Laboratory Technology, College of Science and Technology Hussaini Adamu Federal Polytechnic Kazaure, Kazaure, Jigawa State Nigeria

**Keywords:** Edible vegetable, Heavy metals, AAS analysis, Bioamplification, Risk index

## Abstract

Human exposure to heavy metal toxicity has been linked to the consumption of vegetables grown on polluted soils. The goal of this study was to see how much heavy metals accumulated in the soil, irrigation water, and vegetative sections of lettuce and cabbage planted in Wawan Rafi, Jigawa State, Nigeria. The concentrations of potentially harmful metals in soil, water, and crops are in the order Mn > Pb > Zn > Ni > Cd > Cu, except for lettuce, where Pb was found to be greater than Mn. Furthermore, the majority of the hazardous elements analyzed were below the allowed limit. Nevertheless, the presence of high levels of metals revealed evidence of contamination, which was attributed to human activities. The potential ecological risk index values for lettuce and cabbage are 86.488 and 225.463, respectively, and both are considered safe because the RI values for both lettuce and cabbage are below or within the range of 200 ≤ RI ˂ 400. This implies consumption of these vegetables may not pose a high health risk to the local public when individual heavy metal is considered, but the risk could multiply when all of the heavy metals are considered together.

## Introduction

Environmental pollution due to heavy metals has recently become an emergent ecological catastrophe and serious apprehension as a result of their persistence and non-biodegradability. Consequently, toxic metal pollution becomes the focus of most researchers especially due to the potential impact on the environment and human health as well (Bhatia et al., 2015). Heavy metals reach the soil through a variety of pathways, such as urban, industrial effluents, and aerosols produced by the burning of fossil fuel, smelting of metals, and other anthropogenic processes. Recently, researchers have attributed soil contamination to excessive doses of manure, micronutrients (inorganic) fertilizers, herbicides and insecticides, tannery, mining, and other sources of heavy metal poisoning which have recently been blamed (Bhatia et al., 2015; Emurotu & Onianwa, 2017). Heavy metal contamination of soil can impact negatively on crop physiological development and agricultural yield quality and represent a significant health risk to humans through the food chain (Salazar et al., 2012; Zhao et al., 2014). According to Emurotu and Onianwa (2017), human exposure to toxic metals may be primarily linked to chain transfer from soil to crops. Although some heavy metals are required by plants and other living organisms for normal physiological development, at higher concentrations, they constitute a serious threat (Dessai & Nayak, 2009). Applications of fertilizers, pesticides, compost manures, and polluted water used for irrigational purposes all contribute to a large extent to the rise in soil heavy metal content. Plants cultivated on toxic metal polluted soil subsequently absorb and bioaccumulate the toxic metals in large quantities and eventually compromise food quality and safety (Emurotu & Onianwa, 2017).

Vegetables are edible portions of plants that form an integral part of human daily meals (Uwah & Mkpa, 2016). Vegetables are either consumed raw or cooked with other food condiments. They are regarded as a vital part of human diets due to their supplementary protective food value, as they contain carbs, proteins, vitamins, minerals, and trace elements (Uwah, 2017). Unfortunately, despite their nutritional advantages, leafy greens contribute to heavy metal ingestion through the food chain (Uwah, 2017). Excessive use of inorganic fertilizers and organic manures in the soils where these veggies are cultivated contributes heavily to high amounts of heavy metals uptake in the crops (Uwah, 2017). Hence, heavy metals monitoring in agricultural soils, irrigation water, and food crops has become paramount. Similarly, heavy metal absorption by food crops must be investigated to identify possible health hazards. Lettuce and cabbage are among the vegetables consumed in their raw and cooked state, and they are especially in high demand in the urban areas in Jigawa State. This work, therefore, aims at assessing the concentration level and translocation factor of Pb, Mn, Ni, Zn, Cd, and Cu. Furthermore, the possible ecological risk index of the hazardous metals in the chosen plants will be evaluated.

### Reagents and methods

All reagents were of analytical grade and used without further purification: 65% concentrated HNO_3_, 37% concentrated HCl, and 72% concentrated perchloric acid HClO_4_ were purchased from Sallymore Laboratory. While 99% lead nitrate Pb(NO_3_)_2_, 99.5% zinc oxide (ZnO), 99.0% anhydrous cadmium chloride (CdCl_2_), 98.0% copper nitrate tri-hydrate Cu(NO_3_)_2_.3H_2_O, and 99.0% manganite chloride tetra-hydrate (MnCl_2_.4H_2_O) were purchased from Sigma-Aldrich, nickel nitrate hexa-hydrate Ni(NO_3_)_2_.6H_2_O 99.0% was obtained from Spectrum Chemicals.

### Description of the study area

**Fig. 1:**
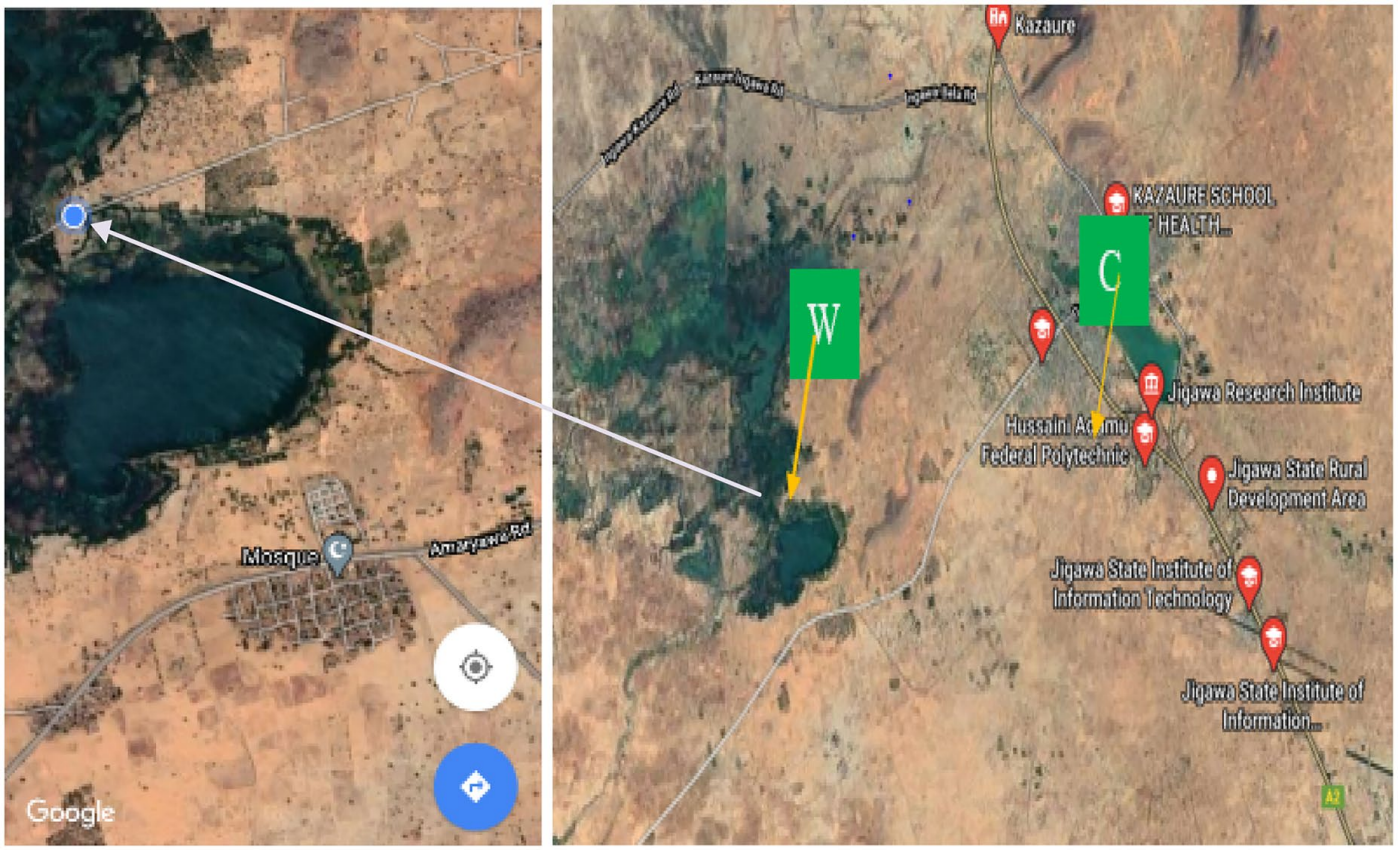
Map of Kazaure Local Government (key: W = Wawan Rafi and C = control site)

The coordinate of Wawan Rafi falls within *12° 39′ 10″ N, 8° 24′ 43″ E* and covered a land area of about 690 sq miles or 1,780 km^2^. The general landform is undulating with dunes, and the area is covered with vast loamy and non-marshy soil that favors agricultural practices. Wawan Rafi is a natural lake surrounded by hills and high land. In addition, runoff water from different points dissolves and transports different minerals, both essential and toxic, and deposits them into the dam. Meanwhile, wastewater from domestic and market areas mostly transports waste to the open lake. Incidentally, this water has found numerous domestic uses including irrigation of vegetable and cereal crops, fishing, and recreational activities among others (http://www.kazaure.net/). The description of the sample site is presented in Fig. 1.

### Soil, plant, and water sampling

The selection of cabbage and lettuce as samples among the vegetables cultivated in the sampling area was mainly based on their availability and the frequency of their consumption. Leaves of the selected vegetable samples were collected from Wawan Rafi river bank in December, January, February, and March. Sampling was achieved using stratified sampling techniques as described by Lawal and Audu (2011). The vegetable samples which were obtained in their prime condition were carefully washed with distilled water and allowed to dry at room temperature, and the edible parts were processed as described in Campos-M and Campos-C (2017). The dry samples were pulverized and the powdered sample was stored in polythene bags for further analysis. For each sampled plant, the surrounding soils were collected using Scoop. The soil samples were dried for 24 h at 40 °C in an oven, then ground, and sieved into fine particles with a pestle and mortar. Representative samples were obtained by coning and quartering technique as described in Onianwa and Fakayode (2000). The coordinates of the sampling location for the vegetable and soil samples are presented in Table 1.

**Table 1:** Sample identification for soil, cabbage, and lettuce samples

Site	Location			Sample coordinates
A	S_D_, S_J_,S_F_ and S_M_	C_D_, C_J_, C_F_ and C_M_	L_D_, L_J_,L_F_ and L_M_	Lat: E008^o^22.200’
				Long: N12^o^37.839’
B	S_D_, S_J_,S_F_ and S_M_	C_D_, C_J_, C_F_ and C_M_	L_D_, L_J_,L_F_ and L_M_	Lat: E008^o^22.286’
				Long: N12^o^37.829’
C	S_D_, S_J_,S_F_ and S_M_	C_D_, C_J_, C_F_ and C_M_	L_D_, L_J_,L_F_ and L_M_	Lat: E008^o^22.209’
				Long: N12^o^37.811’

Key: A, B, and C stand for the three sampling sites. S, C, and L stand for soil, cabbage, and lettuce samples, while D, J, F, and M stand for December, January, February, and March, respectively

The water samples were collected from the dam at depths of 0.5 m, 1.0 m, and 1.5 m, respectively, using a modified Van Dorn sampler, and were transferred to amber-colored polythene bottles. The coordinates of the sampling location for the water samples are presented in Table 2. The temperature and pH of the water samples were recorded immediately and subsequently acidified with 1% HNO_3_ acid to stabilize the metals and conveyed to the laboratory, where they have been filtered through Whatman No 42 filter paper and refrigerated at 4 °C as described in Campos-M and Campos-C (2017).

**Table 2:** Water sample identification

**Site**		**Location/depth (m)**	**Sample coordinates**
A	W_D_, W_J_, W_F,_ and W_M_	0.5	Long: N12 ^o^ 37.622'
			Lat:E008 ^o^ 22.160''
B	W_D_, W_J_, W_F,_ and W_M_	1.0	Long: N12 ^o^ 37.622'
			Lat:E008^o^22.160''
C	W_D_, W_J_, W_F,_ and W_M_	1.5	Long: N12 ^o^ 37.622'
			Lat:E008^o^22.160''

Key: W = Wawan Rafi, the subscript D, J, F, and M stand for December, January, February, and March, while A, B, and C stand for sampling sites

### Digestion of sample

The soil, water, and vegetable samples were digested in a*qua regia*, using a freshly prepared acid mixture of 2 mL 65% HNO_3_ and 6 mL 37% HCl (Jamali et al., 2007; Uddin et al., 2016).

### Digestion of vegetable samples

One gram dried powdered vegetable (cabbage and lettuce) samples were weighed into a separate porcelain crucible and ash-dried in a muffle furnace at 550 °C for 2 h. It is then moved to a separate 100-mL digestion tube, where 9 mL of newly produced aqua regia is added, and the volume is increased to 50 mL with distilled water. The mixture was allowed to gradually boil on a heated plate (at 120 °C) until it was almost dry. The digest was filtered using Whatman No. 42 filter paper and diluted to 50 mL as described in Uddin et al. (2016).

### Digestion of soil samples

Exactly, 1 g each of the soil sample was placed in a 250-mL beaker, followed by the addition of 20 mL freshly prepared *aqua regia*, and the mixture was allowed to boil gently on a hot plate in a fume hood chamber with periodic addition of 10 mL of concentrated nitric acid until the production of red nitrous oxide (NO_2_) ceased. After allowing the sample to cool, 4 mL of 70–72% perchloric acid (HClO_4_) was added and boiled until a clear solution was formed. After cooling, the solution was filtered through Whatman No.42 filter paper and diluted to 50 mL.

### Digestion of water samples

Fifty milliliters each of the sampled irrigation water was taken into a separate 100-mL digestion flask, then 9 mL of freshly prepared *aqua regia* was then added, and the mixture was allowed to evaporate gently to about 20 mL over a hot plate. Then 5 mL of HNO_3_ was added, and the heating was continued until the solution was clear. The digest was filtered using No.42 Whatman filter paper, and the filtrate was diluted to 50 mL.

### Preparation of standards and metal analysis using AAS

The standard stock solutions (1000 mg/L) of each of the metals were prepared from the analytical grade standards while the working solutions were prepared by serial dilutions of the stock solutions and used to obtain the calibration curves. The metals Zn, Cu, Ni, Mn, Cd, and Pb in the soil, water, and vegetable samples were subsequently evaluated in the digested samples. All the metals under investigation were analyzed using Agilent Technologist Atomic Absorption Spectroscopy 240FS (200 series). Equation (1) was then used to compute the metal concentrations in the digested aliquot.

1 $$metal \left(mg/kg\right)=\frac{C \times {V}_{1}}{M}$$

where C represents the concentration in mg/L of the metal being investigated while V_1_ stands for the volume of the final extract which is 50 mL, and M is the mass of the original sample in grams (1 g) (Wang et al., 2019).

### Determination of transfer factor (TF)

Similarly, the transfer coefficient was determined by using Eq. (2) as described by (Kim et al., 2012),

2 $$TF\;or\;{C}_{F}^{i}=\frac{{C}_{plant}}{{C}_{soil}}$$

where C_plant_refers to the metal concentration in plant tissue in mg/Kg of fresh weight and C_soil_ is the metal concentration in soil in mg/Kg of dry weight.

### Potential ecological risk index (RI) of heavy metals:

The RI was calculated using Eqs. (3), (4) and (5);

3 $${F}_{i}=\frac{{c}_{n}^{i}}{{C}_{o}^{i}}$$

4 $${E}_{r}^{i}={T}_{r}^{i}X{F}_{i}$$

5 $$RI={\sum }_{i=1}^{n}={E}_{r Zn+}^{i}+{E}_{r Mn}^{i}+{E}_{r Cu}^{i}+{E}_{r Ni}^{i}+{E}_{r Pb}^{i}+{E}_{r Co}^{i}$$

where ${F}_{i}$ stands for the single metal pollution index; ${c}_{n}^{i}$ is the concentration of metal in the samples; and ${C}_{o}^{i}$ is the metal reference value. The monomial potential ecological risk factor is ${E}_{r}^{i}$ while ${T}_{r}^{i}$ is the metal toxic response factor (Aktaruzzaman et al., 2013). The values for each element are in the order Zn = Mn = 1 < Cu = Ni = Pb = 5 < Cd = 30 (Chen et al., 2020).

## Results and discussion

Tables 3, 4, 5, and 6 reveal the concentrations of the heavy metals (Zn, Mn, Pb, Ni, Cd, and Cu) found in the irrigation water, soils, and vegetables. The results indicated that Zn values in water samples varied from 0.010 mg/L in March to 0.038 mg/L in December, with a mean value of 0.022 ± 0.01 mg/L. However, the recorded Zn levels during the sampling period did not exceed the maximum permissible level in the water (5 mg/L) (Kacholi & Sahu, 2018). The observed low level of Zn in the irrigation water may be attributed to the dilution of the metal in the aquatic medium as described by other researchers (Bhatia et al., 2015). Even though Zn is considered to be an essential micronutrient for human metabolic processes and enzymatic activities (Saper & Rash, 2009), at higher concentrations, however, zinc can be toxic to the living organism (Nazir et al., 2015). This study reveals the concentrations of Zn in soils as 5.405, 2.320, 2.733, and 4.473 mg/Kg for December, January, February, and March, respectively. These values are below the WHO-recommended allowable level of 5–10 mg/Kg for soil (WHO, 1994). Meanwhile, the mean value of Zn concentration recorded for cabbage during the sampling period (6.534 ± 0.38 mg/Kg) was higher than that recorded for lettuce (5.371 ± 1.19 mg/Kg). The Zn metal concentration in the two test plants (lettuce and cabbage) varied, which might be related to differences in their metal uptake capacity as reported by Zurera-Cosano et al. (1989) and Singh et al. (2010).

**Table 3:** Mean concentration of the potentially toxic metals in water and the control (mg/L)

Location	Months	Zn	Mn	Cd	Pb	Ni
WR	Dec	0.038	0.251	0.006	0.071	0.092
	Jan	0.026	0.241	ND	ND	0.223
	Feb	0.014	0.239	ND	0.313	0.328
	Mar	0.010	0.224	ND	0.244	0.084
Control		0.002	0.147	ND	0.052	0.036

Key: *WR* Wawan Rafi, *ND* Not Detected

**Table 4:** Mean concentration of the potentially toxic metals in the soil and the control (mg/Kg)

Mean concentration of the potentially toxic metals in soil sample (mg/Kg)							
Location	Months	Zn	Mn	Cd	Pb	Cu	Ni
WR	Dec	5.405	118.153	0.237	13.910	ND	7.388
	Jan	2.320	99.747	ND	7.400	ND	10.053
	Feb	2.733	62.883	ND	21.098	ND	7.120
	Mar	4.473	100.428	ND	10.330	ND	8.407
Control		1.190	29.995	ND	2.400	2.400	3.315

Key:* WR* Wawan Rafi, *ND* Not Detected

**Table 5:** Mean concentration of the potentially toxic metals in the lettuce and the control (mg/Kg)

Location	Months	Zn	Mn	Cd	Pb	Ni
WR	Dec	6.885	16.050	0.392	11.977	11.263
	Jan	4.105	25.633	0.060	0.803	6.937
	Feb	4.827	10.827	ND	27.082	4.940
	Mar	5.665	13.960	ND	4.288	3.663
Control		2.605	22.125	ND	ND	1.840

Key:* WR* Wawan Rafi, *ND* Not Detected

**Table 6:** Mean concentration of the potentially toxic metals in the cabbage and the control (mg/Kg)

Location	Months	Zn	Mn	Cd	Pb	Cu	Ni
WR	Dec	6.705	12.082	0.573	23.152	ND	10.262
	Jan	6.023	18.767	0.250	5.788	ND	9.955
	Feb	6.498	13.935	ND	28.853	ND	6.022
	Mar	6.908	16.772	ND	13.323	ND	6.188
Control		6.170	22.125	0.300	10.435	1.045	5.600

Key: *WR* Wawan Rafi, *ND* Not Detected

Although plants require only a small amount of Zn for growth and metabolism, all the values recorded in this study exceed the WHO-recommended acceptable maximum of 0.60 mg/Kg. The results of the present work agree with the results previously reported by Ogundele et al. (2015) and Ma and Han (2019).

Manganese is also considered an essential micronutrient necessary in nearly all living organisms. It acts as an enzyme cofactor or a catalytic metal (Andresen et al., 2018). From the results, the manganese concentrations in water samples were found to be 0.251, 0.241, 0.239, and 0.224 mg/L in December, January, February, and March, respectively. These values were found to be higher than the allowed values according to WHO's which is 0.05 mg/L for water (WHO, 1996). Meanwhile, the concentrations of Mn recorded in the soil samples between December and March were: 118.153, 99.747, 62.883, and 100.428 mg/Kg, respectively. These are below the WHO's prescribed limit of 200 mg/Kg. Other researchers recorded Mn concentrations in soils of dumpsites in the range of 90.22 – 318.51 mg/Kg (Abdallah et al., 2011, 2015). For the vegetable samples, the highest manganese concentration recorded was 16.050 mg/Kg in the lettuce sample and 16.772 mg/Kg in the cabbage sample. Manganese has been shown to have a greater tendency to accumulate in unwashed leaves (Okoronkwo et al., 2005). All the values obtained from this analysis are lower than WHO’s suggested permissible limit of 100 mg/Kg. Mn is required in a limited amount for plant growth and reproduction, and it is as important as the other nutrients for development. However, when exposed to high concentrations of Mn, symptoms of toxicity manifest such as hepatic cirrhosis, polycythemia, dystonia, and Parkinson-like symptoms (Alejandro et al., 2020).

Cadmium is a potential carcinogen (Sharma et al., 2016), and Cd-rich wastewaters result in serious human health problems. The "Itai-itai" disease, which was first reported in the 1970s (McLaughlin & Singh, 1999), was the earliest case of Cd toxicity. In this study, Cd was only detected in December in both the soil and water samples with respective concentrations of 0.237 mg/Kg and 0.006 mg/L. In each case, the values were below the stipulated limit of 0.02 mg/L for water and 0.80 mg/Kg for soil. The presence of Cd may be attributed to the precipitation of Cd from aeolian dust during the harmattan season. Interestingly, Cd was only detected in December and January in both lettuce and cabbage samples with concentrations of 0.392 and 0.573 mg/Kg in December and 0.060 and 0.250 mg/Kg in January, respectively. The observed values may also be attributed to aerial depositions during harmattan as reported by Egwu and Agbenin (2013). The values exceeded the WHO's allowable limit of 0.02 mg/Kg. Furthermore, the high concentrations of Cd in the vegetables may also be ascribed to inorganic fertilizers applied to increase yield. The fact that this metal was not detected in the remaining months implies evidence of less anthropogenic activity which contributes to the metal uptake by plants.

Lead is a poisonous metal that is used in significant quantities in many electronic devices. Lead is well known to cause a range of health risks ranging from behavior changes and pedagogical disabilities to seizures and subsequent demise (Sanborn et al., 2002). In the environment, lead may occur from a smelter or battery recycling plant. Lead toxicity can affect renal and neurologic systems as most physicians report that acute lead exposure may lead to anemia which can subsequently lead to heme inhibition and destruction of erythrocytes (Sanborn et al., 2002). In the present study, the highest concentration of Pb was observed in February in both water and soil samples with the recorded values of 0.313 mg/L and 21.098 mg/Kg, respectively. The possible sources of Pb in the environment could be runoffs from waste dumping sites (Abdallah et al., 2015), mechanic workshops (Achi et al., 2011), roads (Okunola et al., 2007), hills surrounding the dams, and atmospheric deposition (Kacholi & Sahu, 2018). Furthermore, lead is the main constituent of lead-acid batteries and tires and can leach and end up in soil (Ogundele et al., 2015). On the other hand, both lettuce and cabbage samples under investigation revealed the highest level of Pb which is 27.082 mg/Kg and 28.853 mg/Kg, respectively, in the same sampling month (February). This result agrees with the findings of Aktaruzzaman et al. (2013). The fact that all Pb concentrations were more than the WHO recommended limit of 2 mg/Kg for vegetable samples suggests that it is not safe to consume these vegetables due to their high-risk potential. The high levels of Pb in the samples may be attributed to the fact that Wawan Rafi River is located few meters away from the Kazaure-Amaryawa road, which to some extent is a busy road with heavy traffic.

Copper is an important microelement for plant growth and development, and it occurs naturally in soil and air. Its content varies depending on soil type and contamination source, and human activities may cause its accumulation above the allowable limit (Onder et al., 2007). In this study, although Cu was among the metals investigated, it was not found in both the water and soil samples. However, 2.400 mg/Kg was detected in the soil control sample which, in comparison, is well below the Dutch normal acceptable maximum level of 36 mg/Kg. A similar observation was reported by Ogundele et al. (2015). The results of the analysis of the two test vegetables (lettuce and cabbage) did not reveal the presence of Cu either.

Nickel acts as an activator of the enzyme urease and is considered an essential trace element for human, animal, as well as plant health. The highest Ni concentration detected in the irrigation water in this study was 0.328 mg/L, which was recorded in February. Meanwhile, the highest Ni concentration (10.053 mg/Kg) observed in the soils was recorded in January. All the values for water samples are higher than the WHO (1996) recommended limit of 0.005 mg/L, but are below 85 mg/kg for soil. This observation suggests that the water may not be fit for domestic, agricultural, or industrial purposes. The results further revealed that the highest Ni concentration 11.263 mg/Kg and 10.262 mg/Kg for lettuce and cabbage, respectively, was recorded in December. These concentrations are above the WHO (1996) maximum tolerable limit of 10 mg/Kg. Nickel metal recorded in other months during the sampling period was, however, below the permissible limit. The fact that cabbage has a higher concentration of Nickel than lettuce suggests that the former may be a stronger accumulator and may further imply its higher toxicity potential. The findings of this study strongly agree with that of Aktaruzzaman et al. (2013) and Hussain et al. (2013) where similar concentrations of Ni were reported in water, soil, and edible parts of vegetables investigated in Mardan District, Pakistan (Aktaruzzaman et al., 2013). Nickel is easily and rapidly absorbed by the plants (Hjortenkrans, 2003), and airborne particles emitted from brakes and tire wear can include considerable quantities of Ni, and in proximity to a busy road may result in a high concentration of Ni in the study samples (Sharma et al., 2016).

### The plant transfer factor

The plant transfer factor is a significant component that is used as a bioindicator of heavy metals in soil. It is also an index that measures metal bioavailability in plants. It has a role in predicting the absorption of such metals by agricultural products (Kim et al., 2012). In this study, the results for the transfer factor of the potentially toxic metals studied are presented in Tables 7 and 8. From Table 7, Cd transfer factor of 1.655 mg/Kg was observed only in December for lettuce, probably indicating evidence of enhanced anthropogenic activity. As anticipated, no transfer factor was observed for Cu, which may be attributed to the fact that Cu was not detected in all the months. For the remaining metals, the highest transfer factors (*TF*) of 1.769 mg/Kg were detected for Zn, followed by 1.525 mg/Kg for Ni, 1.284 mg/Kg for Pb, and 0.257 mg/Kg for Mn. All the transfer factors obtained for lettuce are within the low to moderate contamination factor of $1{\le C}_{F}^{i}<3$ (Ma & Han, 2019).

**Table 7:** Transfer factor of the potentially toxic metals in lettuce sample and the control (mg/Kg)

Location	Months	Zn	Mn	Cd	Pb	Ni
WR	Dec	1.274	0.136	1.655	0.861	1.525
	Jan	1.769	0.257	ND	0.109	0.690
	Feb	1.766	0.172	ND	1.284	0.694
	Mar	1.266	0.139	ND	0.415	0.436
Control		2.189	0.738	ND	ND	ND

Key:* WR* Wawan Rafi, *ND* Not Detected

**Table 8:** Transfer factor of the potentially toxic metals in cabbage sample and the control (mg/Kg)

Location	Months	Zn	Mn	Cd	Pb	Ni
WR	Dec	1.241	0.102	2.423	1.664	1.389
	Jan	2.596	0.188	ND	0.782	0.990
	Feb	2.377	0.222	ND	1.368	0.846
	Mar	1.544	0.167	ND	1.290	0.736
Control		5.185	0.738	ND	4.348	ND

Key:* WR* Wawan Rafi, *ND* Not Detected

Likewise, Table 8 shows that cabbage depicted the highest transfer factor of 2.596 mg/g for Zn, 1.664 mg/g for Pb, 1.389 mg/g for Ni, and 0.222 mg/g for Mn. Again, Cd had a transfer factor of 2.423 mg/Kg which was recorded in December; however, it was not detected for all the other months. Also, no transfer factor was observed for Cu in all the months. The values calculated for cabbage were found to be within the tolerable limit of contamination factor of $1{\le C}_{F}^{i}<3$. Again, in the control sample, Zn and Pb showed 5.185 and 4.348 mg/Kg, respectively, which both fall within the contamination factor values of $3{\le C}_{F}^{i}<6$. The transfer factor and metal concentration in these vegetables are in agreement with the previous studies reported by other researchers (Sharma et al., 2016).

The bioavailability of a metal, soil metal concentrations, metal chemical form, plant absorption capacities, and plant species growth rate all contribute to the transfer factor (Kacholi & Sahu, 2018). The high transfer factor for Zn, Mn, Cd, Pb, and Ni in lettuce and cabbage might be attributable to the heavy metals' greater mobility as a result of natural occurrence, as mentioned in the report of other researchers (Alam et al., 2003). Moreover, anthropogenic activities and the poor retention of these metals to other cations in the soil may contribute to the high transfer factor as well (Kacholi & Sahu, 2018). Furthermore, increasing pollution through wastewater from drainages, solid wastes and sludge applications, solid waste burning, applications of myriad agrochemicals, and vehicular emissions may contribute to high concentrations of these heavy metals (Intawongse & Dean, 2006). Based on the present finding, consumption of the test vegetables may not pose a health danger to the local public when individual heavy metals are considered; however, when all of the heavy metals are taken into account, the risk may be multiplied.

The potential ecological risk index (RI) indicates the degree of heavy metal pollution; it also evaluates the ecological risk caused by toxic metals in the environment (Qiu, 2010). From Table 9, the RI was 86.488 for lettuce and 225.463 for cabbage, with respective RI values of 5.702 and 36.109 for the control sample. This indicates that lettuce has a lower RI value which can be classified as low risk, and cabbage is considered a considerable risk, but both are safe for consumption (Aktaruzzaman et al., 2013).

**Table 9:** Potential ecological risk index (IR) of the potentially toxic metals at Wawan Rafi and the control

Sample I.D	Lettuce	Grade of the environment	Cabbage	Grade of the environment
WR	86.488	Low risk	225.463	Considerable risk
Cont	5.702	Low risk	36.109	Low risk

Key:* WR* Wawan Rafi, *ND* Not Detected

## Conclusion

In this study, the level of Pb, Cd, Zn, Cu, Mn, and Ni was investigated in soil, irrigation water, and vegetative parts of lettuce and cabbage in Wawan Rafi, Kazaure Local Government Area, Jigawa State, Nigeria. The trend in the toxic metal concentrations analyzed in the soil sample is in the order: Mn > Pb > Zn > Ni > Cd > Cu while the trend in the concentration of the metals in water is: Ni > Pb > Mn > Zn > Cd > Cu. Some of the potentially toxic metals were within the allowable limits, whereas others exceeded the limits, except for Cu which is below the detection limit as observed. All the samples analyzed contain significant levels of these metals, which indicated evidence of metal pollution in the study area, and this may be ascribed to high levels of vehicular emissions as well as other anthropogenic activities along the roads bordering the sampling area. Pollution from nonpoint sources such as residential runoffs, offices, and other commercial facilities, as well as industrial discharge, chemical smokestacks, and urban sewage, may also contribute to the heavy metal load in the sampling area. Therefore, the study recommends continuous monitoring of the potentially toxic metals present in irrigation water, soil, and vegetables in the sampling area to minimize the accumulation of the metals and thus prevent human health hazards.

## Declarations

### Data availability

The authors confirm that all data gathered or analyzed during this study are included in this published article.
